# Probes for the heterogeneity of muscimol binding sites in rat brain

**DOI:** 10.3389/fphar.2024.1368527

**Published:** 2024-03-14

**Authors:** Veronika Müller, Margot Ernst, Aygul Baykuchkarova, Filip Koniuszewski, Konstantina Bampali, Thomas Seidel, Petra Scholze

**Affiliations:** ^1^ Department of Pathobiology of the Nervous System, Center for Brain Research, Medical University of Vienna, Vienna, Austria; ^2^ Department of Pharmaceutical Sciences, Division of Pharmaceutical Chemistry, University of Vienna, Vienna, Austria

**Keywords:** GABAA-receptor subtypes, radioligand displacement analysis, muscimol binding, rat brain, binding site heterogeneity

## Abstract

**Introduction:** The plant-based alkaloid muscimol is a potent agonist of inhibitory GABA_A_-neurotransmitter receptors. GABA_A_ receptors are a heterogeneous family of pentameric complexes, with 5 out of 19 subunits assembling around the central anion pore. Muscimol is considered to bind to all receptor subtypes at the orthosteric drug binding site at the β+/α− interface. Recently, we observed that the antipsychotic drugs clozapine (CLZ), loxapine (LOX) and chlorpromazine (CPZ) although exerting functional inhibition on multiple GABA_A_ receptor subtypes showed diverging results in displacing 3H-muscimol. While a complete displacement could be observed in hippocampal membranes by bicuculline (BIC), and no displacement with CPZ, the compounds CLZ and LOX competed partially. Non-sigmoidal, complex dose response curves were indicative of multiple sites. In the current study we now aimed to investigate more extensively this heterogeneity of bicuculline sensitive muscimol sites in rat brain.

**Methods:** We tested membranes from four different brain regions (hippocampus, cerebellum, thalamus and striatum) and selected recombinantly expressed subunit combinations with displacement assays. 3H-muscimol displacement was tested with BIC, LOX, CLZ and CPZ. *In silico* ligand structural analysis and computational docking was performed.

**Results:** We observed a unique pharmacology of each tested compound in the studied brain regions. Combining two of the tested ligands suggests that in striatum all CLZ sites are contained in the pool of LOX sites, while the CPZ sites may in part be non-overlapping with LOX sites. Experiments on recombinantly expressed receptors indicate, that BIC can displace 3H-muscimol from all tested receptors, while LOX and CLZ display different and variable competition indicative of multiple sites. Molecular docking produced structural correlates of the observed diversity of muscimol sites on the basis of bicuculline bound experimental structures.

**Discussion:** These findings indicate that 3H-muscimol binding sites in rat brain are heterogeneous, with different populations of receptors, which are CPZ, LOX or CLZ sensitive or insensitive. These binding sites show a varying distribution in different rat brain regions. Molecular docking suggests that the so-called loop F region of α subunits drives the observed differences.

## 1 Introduction

GABA_A_ receptors are pentameric ligand gated ion channels, which in mammals assemble from 5 out of 19 subunits and their respective variants (splice isoforms, RNA-editing variants, etc.) (Olsen and Sieghart, 2008; Brickley and Mody, 2012). Homology modeling studies, experimental structure analysis as well as mutational analysis have given us insight into the fact that those receptors contain multiple different ligand binding sites, which allow pharmacological receptor modulation. Among them are the orthosteric GABA binding site, but also the allosteric interaction sites for benzodiazepines, pyrazoloquinolinones, barbiturates, Zn2+, as well as regions binding inhibitors such as picrotoxinin, and also interaction sites for many other compounds (Sieghart, 2015; Puthenkalam et al., 2016; Fabjan et al., 2021). The psychoactive alkaloid muscimol isolated from the mushroom Amanita muscaria, has long been identified as a structural analogue of the neurotransmitter GABA, binding to the orthosteric GABA binding site. It exerts a strong GABA-like activity on neurons, however, it serves only as a weak non-competitive inhibitor of GABA uptake mediated by GABA transporters (Johnston, 1971). This selectivity has made 3H-muscimol an ideal candidate to be one of the first radioligands other than 3H-GABA to study GABA_A_-receptors (Snodgrass, 1978) and has been used in multiple studies ever since. Muscimol has always been regarded to be a universal agonist for all GABA_A_-receptor subtypes independent of the nature of the subunits (Sieghart, 1995; Mehta and Ticku, 1999). This knowledge has recently been questioned after studying several different GABA_A_ receptor knock-out mice. α4- as well as δ-knock out mice show reduced high-affinity 3H-muscimol binding in several brain regions, such as thalamus or hippocampus, while there was no significant reduction in 3H-muscimol binding to brains from α1-knock-out animals, indicating a complex diversity of 3H-muscimol binding sites (Mihalek et al., 1999; Korpi et al., 2002; Chandra et al., 2010).

In a previous study from our lab (Bampali et al., 2022) we analyzed several different, clinically frequently used antipsychotic and antidepressant medications for their effects on binding to and modulating several GABA_A_ receptor subtypes. We used 3H-muscimol displacement experiments to complement our two-electrode voltage-clamp electrophysiological analysis on *Xenopus laevis* oocytes (see Figures 6e and S11 of Bampali et al., 2022). While all studied antipsychotic compounds exerted functional inhibition on multiple GABA_A_ receptor subtypes, our radioligand displacement data indicated that from our selection of compounds only clozapine (CLZ), loxapine (LOX) and bicuculline (BIC) interacted directly with the orthosteric GABA binding site. Chlorpromazine (CPZ), although able to functionally modulate GABA_A_ receptors, could not displace 3H-muscimol from hippocampal membranes, obviously interacting with a yet unidentified additional binding site. Interestingly, while a complete displacement could be observed by BIC, the other two compounds CLZ and LOX could only incompletely compete with the radioligand, leading to a maximal displacement of 46% and 71% respectively. In addition, the dose-response curves of those three ligands displayed a non-sigmoidal complex form, clearly indicating that they are not following a standard one-site binding pharmacology. In the current study we now investigated the pharmacology of 3H-muscimol displacement by BIC, CLZ, LOX and CPZ more closely, focusing on multiple different brain regions and trying to elucidate the background behind the observations of a complex pharmacology.

## 2 Materials and methods

### 2.1 Preparation of rat brain membranes

Female rats (3–4 weeks old) were sacrificed by decapitation and the brains removed quickly. The desired brain regions (cerebellum, hippocampus, striatum and thalamus) were dissected, flash frozen in liquid nitrogen and stored at −80°C until needed. Ethical review and approval for this procedure was not required because the EU directive 210/63/EU, which is also reflected by the Austrian federal law “Tierversuchsgesetz 2012,” states that sacrificing of animals solely for the use of their organs and tissues is not considered a “procedure” and does not require specific approval. Brain tissue was homogenized with an Ultra-Turrax rotor-stator homogenizer for 30 s in ice-cold homogenization buffer (10 mM Hepes, 1 mM EDTA, 300 mM Sucrose) and centrifuged at 45,000 g at 4°C for 30 min. The pellet was resuspended in wash buffer (10 mM Hepes, 1 mM EDTA), incubated on ice for 30 min and centrifuged at 45,000 g at 4°C for 30 min. The pellet was stored at −80°C o/n and the next day washed three times by suspension in 50 mM Tris-citrate buffer, pH = 7.1 and subsequent centrifugation as described above. Membrane pellets were stored at −80°C until final use.

### 2.2 Culturing of human embryonic kidney HEK-293 cells

Human embryonic kidney HEK-293 cells (American Type Culture Collection ATCC^®^ CRL-1574TM) were maintained in Dulbecco’s modified Eagle medium (DMEM, high glucose, GlutaMAX^®^ supplement, Gibco 61965-059, ThermoFisher, Waltham, Massachusetts, USA) supplemented with 10% fetal calf serum (Sigma-Aldrich F7524, St. Louis, Missouri, USA), 100 U/mL Penicillin-Streptomycin (Gibco 15140-122, ThermoFisher, Waltham, Massachusetts, USA) and MEM (Non-Essential Amino Acids Gibco 11140-035, ThermoFisher, Waltham, Massachusetts, USA) on 10 cm cell culture dishes (Cell+, Sarstedt, Nürnbrecht, Germany) at 37°C and 5% CO2.

HEK-293 cells were transfected with cDNAs encoding rat GABA_A_ receptor subunits subcloned into pCI expression vectors using the FectoPRO^®^ (Polyplus) transfection kit. 3 Mio cells were seeded into a 10 cm cell culture dish containing 9 mL culture medium 1 day prior to transfection. 5 μg of DNA at a ratio of α:β = 1:1 were diluted in 1 mL serum-free media, added to 10 µL FectoPRO^®^ reagent and incubated for 10 min at RT. The mixture was pipetted to the cells and 5 µL FectoPRO^®^ Booster added. Cells were harvested 24–36 h after transfection by scraping into phosphate buffered saline. After centrifugation (10 min, 3,000 g, 4°C) cells were resuspended in TC50 (50 mM Tris-Citrate pH = 7.1), homogenized with an Ultra-Turrax^®^ (IKA, Staufen, Germany), centrifuged (10 min, 3,000 g, 4°C) and the pellet was frozen at −20°C until needed.

### 2.3 Radioligand membrane displacement assays

Frozen membranes were thawed, resuspended and incubated for 60 min at 4°C in a total of 400 µL of TC50/NaCl (50 mM Tris-Citrate pH = 7.1; 150 mM NaCl), various concentrations of the drug to be studied and 10 nM 3H-muscimol in the absence or presence of 10 mM GABA (to determine unspecific binding; final DMSO-concentration 1%). Membranes were filtered through Whatman GF/B filters and the filters were rinsed twice with 4 mL of ice-cold 50 mM Tris/citrate buffer. Filters were transferred to scintillation vials and subjected to scintillation counting after the addition of 3 mL Rotiszint HighCapacity liquid scintillation cocktail (Carl Roth, Germany) using a TriCarb 4910TR from Perkin Elmer.

### 2.4 Data analysis

The individual data points of full dose-response curves were measured in duplicates and repeated in 3-4 independent experiments. Curves were analyzed using GraphPad Prism version 10.1.1. for Mac OS X and the equation “log(inhibitor) vs. response” with Y=Bottom + (Top-Bottom)/(1 + 10^((LogIC50-X)*HillSlope). The Top and the Hillslope were restrained to 100% and −1 respectively, the Bottom and the IC50 left variable.

For the comparison of the degree of ligand displacement at 1mM, 3-9 independent measurements were performed in triplicates each. One-way ANOVA followed by Tukey’s multiple comparisons test was performed using GraphPad Prism version 10.1.1. for Mac OS X with ns = p> 0.05, *p < 0.05, **p < 0.01, ***p < 0.001 and ***p < 0.0001.

### 2.5 Ligand preparation for docking analysis

The molecular structures of bicuculline (BIC), loxapine (LOX), clozapine (CLZ) and chlorpromazine (CPZ) were downloaded from the DrugBank website https://go.drugbank.com (Wishart et al., 2018) in SDF-format. Afterwards, protonation states of nitrogen atoms were adjusted for pH 7.4 according to their pKa values calculated by means of MarvinSketch (https://docs.chemaxon.com/display/docs/pka-plugin.md , V17.27.0; default settings). Finally, for each ligand a conformer ensemble was generated using the software CONFORGE V1.0.0 (Seidel et al., 2023). CONFORGE was executed with the following settings: max. output ensemble size = 200 (-n option), energy window = 20.0 kcal/mol (-e option), and RMSD threshold = 0.25 Å (-r option). All other settings were left at their default values and the generated conformer ensembles were saved in a single multi-conf. SDF-file for further processing.

### 2.6 Shape-based alignment

The shape-based alignment of LOX, CLZ, CPZ and BIC (for control purposes only) to the binding pose of BIC in the investigated complexes with PDB (Berman et al., 2000) codes 6HUK and 6X3S was carried out using of the software ROCS (http://www.eyesopen.com) (Hawkins et al., 2007) (V3.4.1.0). The bound-state conformations of BIC in 6HUK (chain E) and 6X3S (chain A) that served as query structures for the subsequent ROCS runs were first extracted from the complexes by means of LigandScout (https://www.inteligand.com/ligandscout3/) (Wolber and Langer, 2005) (V4.4.8) and saved in SDF-format. Afterwards, for each bound-state BIC pose a ROCS run using the previously prepared ligand multi-conf. SD-file as screening database was carried out. ROCS was executed with the following settings: -mcquery false, -maxhits 5000, -oformat sdf, -report one, -status none. All other settings were left at their default values. The thus obtained hit output files then contained the best scoring (ranked by TanimotoCombo) alignments of LOX, CLZ, CPZ and BIC to the respective bound-state BIC conformation.

### 2.7 Docking

Docking of LOX, CLZ, CPZ into the BIC binding-site of 6HUK (chain E) and 6X3S (chain A) was carried out in the structure-based perspective of LigandScout using LigandScout’s built-in AutoDock Vina XBSF module (Koebel et al., 2016). The LOX, CLZ, and CPZ alignment poses that were obtained by the ROCS screening runs for each of the complexes served as corresponding input structures. Each docking run was carried using LigandScout’s default settings for AutoDock Vina XBSF and the generated poses were saved in SDF-format for further processing.

## 3 Results

**FIGURE 1:**
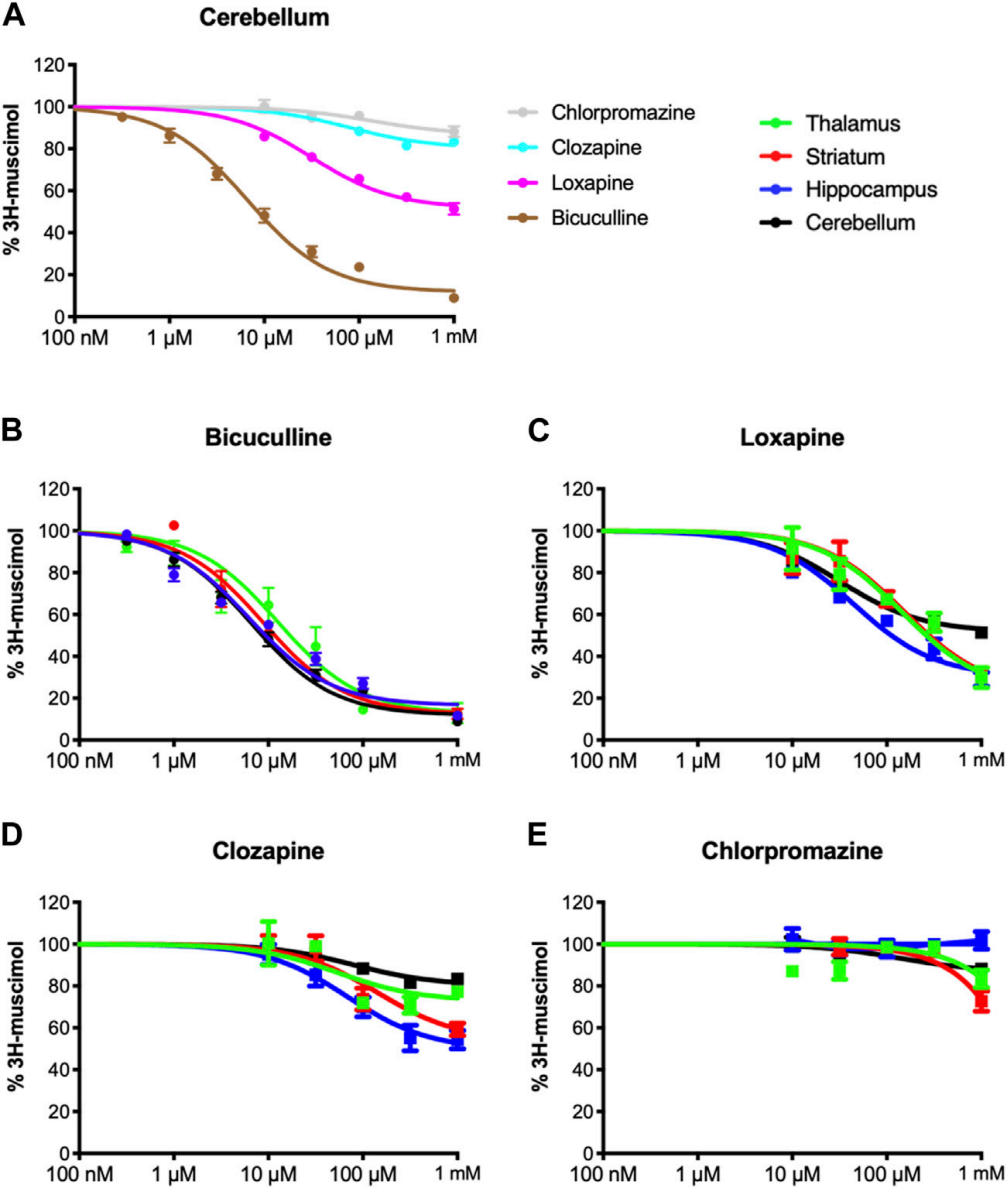
Inhibition of 3H-muscimol-binding to rat brain membrane GABA_A_ receptors. Membranes were incubated with 10 nM 3H-muscimol in the presence of various concentrations of the displacing ligand. 100% is the amount of radioligand bound in the presence of 1% DMSO. Data shown are mean ± SEM of 3-9 independent experiments performed in duplicates each. **(A)**: displacement of 3H-muscimol binding to cerebellar membranes by the four test compounds. **(B–E)**: 3H-muscimol binding experiments on membranes of four different brain regions (cerebellum, hippocampus, thalamus and striatum) incubated with varying concentrations of BIC, LOX, CLZ or CPZ.

**FIGURE 2:**
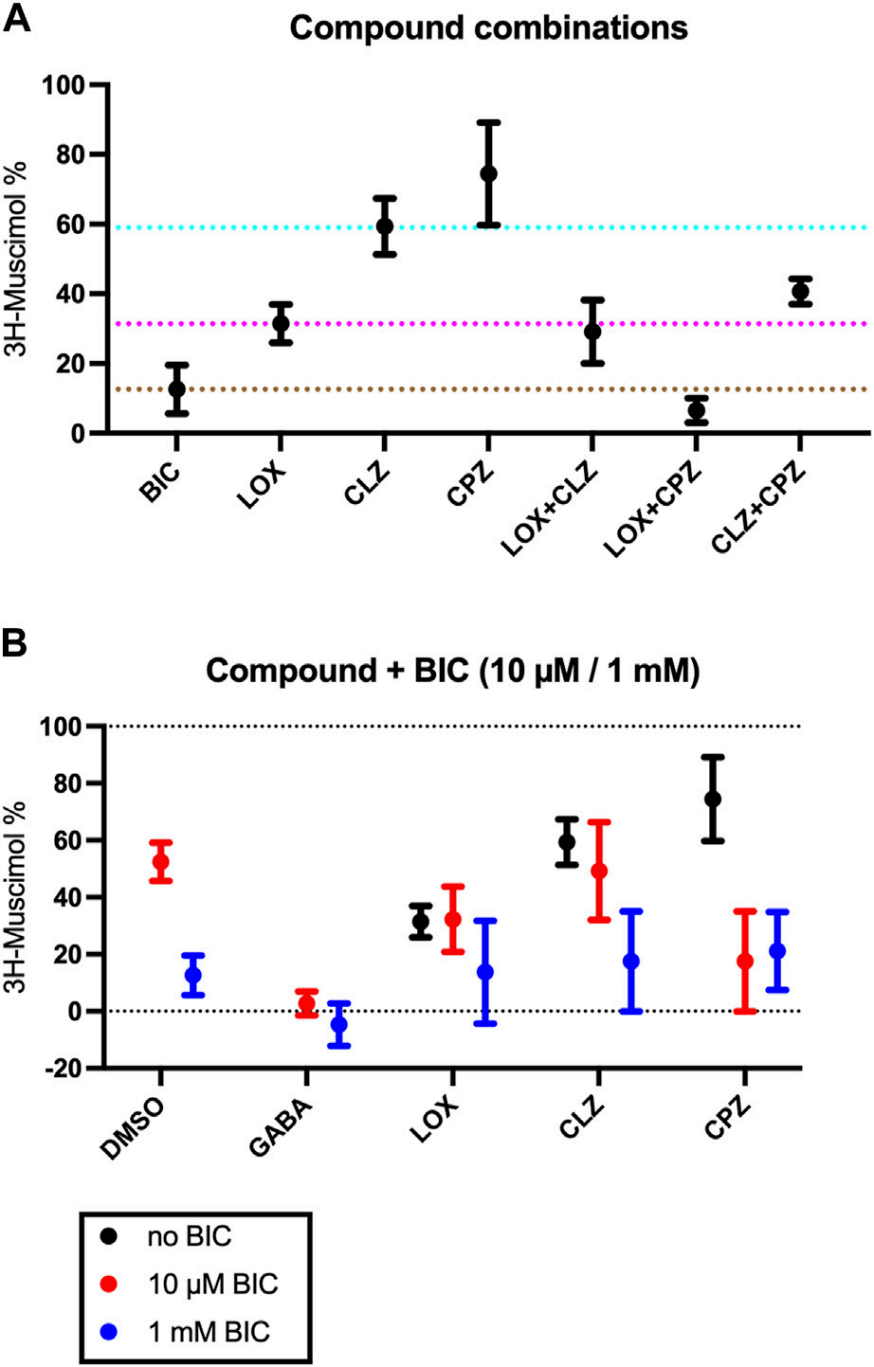
Effect of co-application of two study compounds. Rat striatal membranes were incubated with 10 nM 3H-muscimol together with 1 mM BIC, 1 mM LOX, 1 mM CLZ or 1 mM CPZ or a combination of those drugs. The color of the dotted lines match the colors from Figure 1A (Panel**(A)**) . Membranes were incubated with 10 nM 3H-muscimol together with10 µM or 1 mM BIC (red or blue symbols in Panel **(B)**, respectively) combined with 1 mM of LOX, 1 mM CLZ or 1 mM CPZ. Data shown are mean ± SEM of 4 independent experiments performed in duplicates each.

Inspired by our previous findings of incomplete 3H-muscimol displacement by several antipsychotic drugs in hippocampal membranes (Bampali et al., 2022), we studied cerebellar membranes, which have been investigated previously for CLZ (Korpi et al., 1995). In Figure 1A we show 3H-muscimol displacement in cerebellar membranes by BIC, LOX, CLZ and CPZ with the protocol as or study by Korpi et al. (1995), displacement by CLZ is less in cerebellar membranes compared to hippocampal membranes. In contrast, CPZ which did not displace 3H-muscimol from hippocampal membranes (Bampali et al., 2022), displaced in the cerebellar membranes by 14%. Intrigued by these differences between hippocampal membranes and cerebellar membranes, we investigated thalamus and striatum in addition. Both of them are rich in α4-containing GABA_A_-receptors (Pirker et al., 2000), which have been associated with high-affinity muscimol binding sites (Chandra et al., 2010). Figures 1B–E show the 3H-muscimol binding experiments on membranes of four different brain regions (cerebellum, hippocampus, thalamus and striatum) incubated with varying concentrations of BIC, LOX, CLZ or CPZ. In the Supplementary Figure S1 the statistical analysis of the 3H-muscimol displacement at 1 mM of each drug is shown. The degree of displacement is close to saturation at 1 mM of all compounds as evidenced by the flattening of the displacement curves, thus, the differences in total displacement reflect different pool sizes of sites sensitive to the individual compounds. Although each of the brain regions shows a unique displacement profile, in all tested areas the rank order of the tested ligands is consistently BIC > LOX > CLZ > CPZ. CPZ displaced 3H-muscimol in cerebellum and striatum, with the values being significantly different from 100% (no displacement), as estimated by a one sample t and Wilcoxon test. The effect is most prominent in striatum with a displacement of 35%. We therefore decided to focus on studying the striatum more closely and to investigate whether the compounds bind to partly overlapping sites. As can be seen in Figure 2A with the statistical analysis shown in the Supplementary Figure S2, a combined drug application of LOX and CLZ gave similar results as LOX alone (to the level of the magenta dotted line in Figure 2A). In contrast, a combination of LOX and CPZ reduced 3H-muscimol binding almost to background levels, exhibiting significantly more displacement than either of the two drugs alone (to the level of the brown dotted line in Figure 2A). The data with the ligand combinations therefore suggest that all CLZ sites are contained in the pool of LOX sites, while the striatal CPZ sites appear to be non-overlapping and thus additive with LOX sites. This implies that CLZ and CPZ should also be additive. A close look at Figure 2A shows that the displacement by the combination is larger than CLZ alone (blue dotted line in Figure 2A), but this difference in not significant (Supplementary Figure S2).

The observed muscimol displacement could be fully competitive, or partly allosteric. In order to investigate if CPZ (or any of the other study compounds) could influence BIC binding allosterically, and might possibly be able to shift BIC-dose response curves, we investigated the effect of maximal concentrations (1 mM) of LOX, CLZ and CPZ together with 10 µM or 1 mM BIC (Figure 2B, red and blue symbols, respectively). 10 μM BIC leads to a half-maximal displacement of 3H-muscimol. Combining 1 mM of any of the compounds with 1 mM BIC results in displacements that are not significantly different from 1 mM BIC alone (blue symbols in Figure 2B). The displacement by LOX is not further increased by 10 µM BIC, the CLZ displacement is not significantly increased by the addition of 10 µM BIC, but the displacement by the combination of CPZ with 10 µM BIC displays the expected increase of the total displacement. These findings suggest that none of the study-compounds has a significant allosteric effect and none of them is able to shift the BIC dose response-curves significantly to either side, however, the lacking additive effects that would be expected for saturating LOX or CLZ together with half maximal BIC would be small and may be masked by experimental scatter. Thus small allosteric effects beyond the orthosteric competition cannot be completely excluded.

**FIGURE 3:**
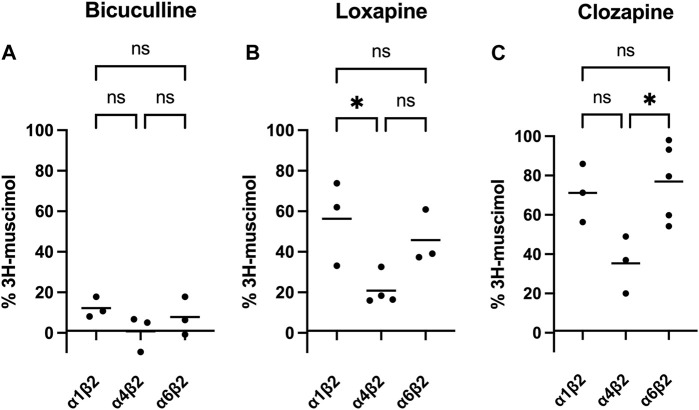
Inhibition of 3H-muscimol-binding to recombinantly expressed GABA_A_ receptors, by BIC (panel **(A)**), LOX (panel **(B)**) and CLZ (panel **(C)**). HEK-293 cells were transfected with selected GABA_A_-receptor subunits, and the membranes were incubated with 10 nM 3H-muscimol in the presence of 1 mM of drug. 100% is the amount of radioligand bound in the presence of 1% DMSO. Data shown are means of 3–5 independent experiments performed in triplicates each. One-way ANOVA followed by Tukey’s multiple comparisons test was performed to determine statistically significant differences between receptor subtypes, where ns = *p* > 0.05, *p < 0.05, **p < 0.01 and ***p < 0.001.

Of course, it is tempting to speculate, that the observed heterogeneity is based on different affinities of the four test compounds to different GABA_A_-receptor subtypes. While cerebellum only expresses a limited set of the 19 GABA_A_-receptor subunits (α1, α6, β2, β3, γ2 and δ), the other brain regions tested are much more complex. Though lacking α6, among other subunits large populations of α5-or α4-containing GABA_A_ receptors are expressed in hippocampus and striatum or thalamus, respectively (Pirker et al., 2000). Especially α4-containing receptors caught our attention, since experiments on α4-knock-out mice showed reduced high-affinity 3H-muscimol binding in several brain regions (Chandra et al., 2010). We therefore transfected HEK-293 cells to express three different subunit combinations: α1β2, α4β2, and α6β2. α1-containing receptors are being expressed ubiquitously in most brain regions, the subunit α6 is specifically found in cerebellum, and α4 is enriched in thalamus and striatum (Pirker et al., 2000). Although the most prominent receptors in the brain are α1-and γ2 subunit containing (Olsen and Sieghart, 2008; Sun et al., 2023), binary αβ-receptors have been shown to co-exist with classical GABA_A_-receptors, although in low numbers (Brickley et al., 1999; Mortensen and Smart, 2006). It is well known that in heterologous expression systems complex receptors formed from three or more subunits might assemble insufficiently and/or with multiple subunit arrangements, which complicates data interpretation (Ebert et al., 1996; Baumann et al., 2001). We therefore decided to minimize the number of co-transfected subunits to αβ only, an approach that has been applied previously by us and others, especially when studying α+/β- or β+/α-interfaces (Ramerstorfer et al., 2011; Varagic et al., 2013; Che Has et al., 2016). Membranes from recombinantly expressed α1β2, α4β2 and α6β2 combinations were incubated with 3H-muscimol and 1 mM of BIC, LOX or CLZ (see Figure 3). While BIC potently competed for 3H-muscimol in all of the three tested subunit combinations, LOX and CLZ displaced the radioligand to about 20%–30% residual binding in α4β2, but only to 60%–70% residual binding in α1β2 or α6β2. The incomplete displacements and the variability compared to bicuculline indicate an unexpected heterogeneity of sites in recombinantly expressed subunit combinations. The unexpected variability prompted us to not further use this expression system.

**FIGURE 4:**
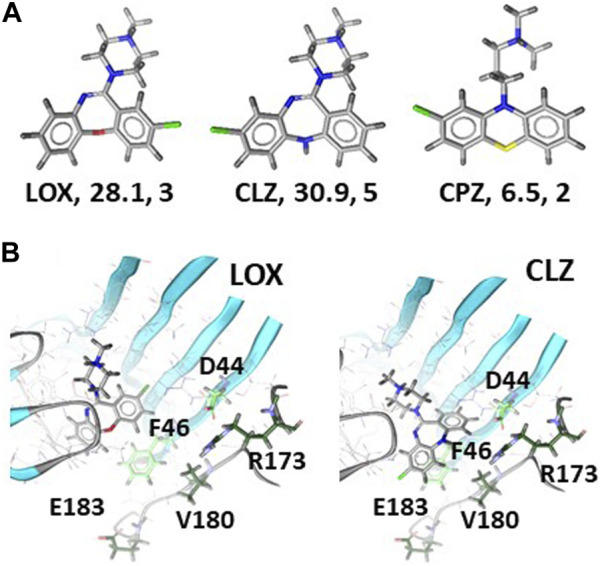
Ligand properties and docking of LOX and CLZ to 6HUK: Panel **(A)** 3D rendering, polar surfaces in Å^2^, number of available features for each ligand. Panel **(B)** Top ranked poses of LOX (left) and CLZ (right). Amino acids of the α1 subunit that potentially contribute to differential affinities are displayed as green sticks and labeled. Light green sidechains are within 6Å of BIC in the experimental structure, dark green ones within 8 Å. Supplementary Figure S4 provides the homologous amino acids for the other α isoforms.

Next, we aimed to find structural correlates of the observed diversity of muscimol sites that are present in the samples from brain tissue and recombinantly expressed GABA_A_ receptor subunit combinations. We first analyzed the ligands and asked whether a ligand-based approach would provide any correlates for the observed rank order of the pool sizes for muscimol sites that are accessible to the test ligands. The ligands were subjected to shape and feature analysis, and the overlap with BIC was examined, see Figure 4A and Supplementary Figure S3. LOX and CLZ feature similar polar surfaces, while CPZ has much less polar surface and fewer directional features (H- bonding abilities in this case). All three molecules feature an ionizable amino group, as does BIC. The best shape alignment for all three test compounds features very close overlap with BIC, with the tricyclic core occupying the same pocket volume close to the complementary subunit, and the ionizable group behind and below segment C. Based on this analysis, it can be expected that LOX and CLZ behave similar with respect to their ability to occupy GABA/bicuculline pockets, which are fairly polar and only a moderate degree of selectivity as they are in contact with the conserved pocket regions. CPZ would be expected to be generally less potent due to the pronounced hydrophobicity and lower number of available interaction features. In agreement with the prediction of low potency, the experimental data strongly suggest that CPZ is unlikely to recognize β2,3/α1 muscimol sites which should be the most abundant ones in most rat brain regions.

In contrast to the predicted similarity between LOX and CLZ by the ligand based approach, the experimental results imply that CLZ cannot interact with all LOX sensitive muscimol sites. We thus turned to computational docking to further search for structural hypotheses that reflect differences between LOX and CLZ bound complexes. Two bicuculline bound cryo-EM structures are available at this time, namely 6HUK (Masiulis et al., 2019) with the β3+/α1-pocket, and 6X3S (Kim et al., 2020) with the β2+/α1-pocket. These represent two of the possible 18 β+/α-interfaces. BIC, in line with its unselective binding properties, adopts virtually the same binding mode in these two interfaces. Sequence-to-structure alignments of the remaining 16 possible interfaces indicates that the principal subunit contributes only one variable position on segment C to the bicuculline pocket, while the complementary subunit features two variable positions in direct contact, and three more on segment F within a short distance thereof, see Supplementary Figure S4. Docking of LOX and CLZ into both available bicuculline pockets yielded a surprising diversity of high-ranking poses. The top-ranking LOX pose in the 6HUK model occupies the same volume as BIC, displays well saturated interaction features and places the molecule in proximity to the conserved portions of the BIC pocket, see Figure 4B. Thus, it is a plausible candidate and consistent with the observation that LOX displaces a large fraction of muscimol in the tested brain regions, thus, coming close to the unselective action of BIC. The top ranked pose closely overlaps with the LOX top ranked pose, and thus would suggest overlapping pharmacological properties. The rank 2 pose – with an identical estimated binding energy – features less overlap with BIC and the tricyclic moiety in closer proximity to the variable parts of the complementary subunit. Thus, this pose is consistent with some degree of α isoform selectivity, see Figure 4B. A similar pose is also found in rank 3 of the 6X3S posing space, which was otherwise inconclusive. Even more support for this pose comes from the roughly similar placement of the ionizable group relative to BIC. Thus, computational docking provides a testable structural hypothesis for potential binding of LOX and CLZ to the β3+/α1-pocket and a plausible correlate for more pronounced selectivity of CLZ due to the close proximity of CLZ to variable “loop F” residues. Due to lacking structures for other α isoforms, it is not yet possible to predict which pocket can accommodate LOX and CPZ in the subtypes present in rat brain.

## 4 Discussion

In this study we examined the sensitivity of muscimol binding sites in four brain regions to different agents, on the assumption of a simple competitive mechanism. The psychoactive alkaloid muscimol isolated from the mushroom Amanita muscaria, has long been identified as a structural analogue of GABA that also binds to the orthosteric β+/α- GABA binding site (Smith and Olsen, 1994). However due to the existence of six different α and three different β subunits (Sieghart, 2015), there is not one single β+/α-interface, but 18 of them. Additional non-canonical sites might exist that involve other subunit interfaces, such as β3+/δ- (Baur et al., 2009; Kaur et al., 2009; Lee et al., 2016) or β2+/β2- (Wongsamitkul et al., 2017). So far it has always been assumed, that muscimol binds to all β+/α-interfaces independent of the nature of the subunits (Chandra et al., 2010) and that the antagonist bicuculline is equally unselective. Interestingly, there was no significant reduction in 3H-muscimol binding to brains from α1-knock-out animals, while for the less abundant α4-as well as δ-subunits knock out mice show reduced high-affinity 3H-muscimol binding in several brain regions, such as thalamus or hippocampus (Mihalek et al., 1999; Korpi et al., 2002; Chandra et al., 2010). The observation that in different knock-out mice, not all subunits lead to the expected loss of muscimol sites, raises the question whether individual interfaces could be detected individually with subtype selective agents.

Here we now describe differential muscimol displacement by three tricyclic ligands clozapine, loxapine and chlorpromazine, compared to bicuculline. Those compounds are well known frequently prescribed antipsychotics, which elicit their effect mostly by targeting dopamine as well as serotonin receptors (Mauri et al., 2014), and have been suggested to also mediate at least part of their action by targeting GABA_A_ receptors (Squires and Saederup, 1997, 1998; Bampali et al., 2022). Our results suggest that those three compounds not only interact with GABA_A_ receptors but can also distinguish between different receptors containing different muscimol sites. In all tested brain regions, we see less displacement by the test compounds compared to bicuculline at nearly saturating concentratons. The compound ranking is also the same in all brain regions, with the amount of chlorpromazine sensitive sites being lowest, followed by clozapine, and finally loxapine with the largest pool of sites in all tested brain regions.

As a clear indication for differences not only in the number of available sites but also for somewhat different affinities, we observed non-sigmoidal displacement curves in hippocampus, striatum and thalamus, while sigmoidal curves are seen for cerebellum. This supports the notion that chiefly the diversity in α subunits drives the affinity of the sites for the test molecules, because the cerebellum contains only two α isoforms in appreciable amounts (Pirker et al., 2000; Scholze et al., 2020). Generally, a limited subset of 6 out of 13 GABA_A_-receptor subunits (α1, α6, β2, β3, γ2 and δ) was found in cerebellum (Pirker et al., 2000), and therefore only selected receptor subtypes are being expressed (Jechlinger et al., 1998; Poltl et al., 2003). In contrast, hippocampus, striatum and thalamus, though lacking α6, among other subunits express large populations of α5-or α4-containing GABA_A_ receptors (Pirker et al., 2000) forming a huge repertoire of receptor subtypes with putative differing pharmacology. Especially loxapine-sensitive receptors are less abundant in cerebellum (leading only to about 50% of 3H-muscimol displacement), while in hippocampus, thalamus and striatum significantly more receptors are targeted by loxapine. Chlorpromazine also interacts with some muscimol binding sites on the GABA_A_ receptors, but consistently fewer compared to loxapine and clozapine. In hippocampus we saw no competition, in cerebellum small, but significant amounts of 3H-muscimol could be displaced, and in striatum we even found an effect of competing for 35% of the binding sites. Combining two of the tested ligands suggests that in striatum all clozapine sites are contained in the pool of loxapine sites, while the chlorpromazine sites may in part be non-overlapping with the loxapine sites. In order to test the notion that all displacement occurs competitively, we combined nearly saturating concentrations (1 mM) of LOX, CLZ and CPZ with half-maximal and saturating BIC, and observed no evidence for allosteric (non-competitive) interactions. Thus, while allosteric effects cannot be excluded with certainty, the data suggests a simple competitive mechanism of muscimol displacement for the four compounds.

We aimed to more closely identify the receptor subtypes, which might be responsible for the observed heterogeneity, assuming different affinities of the four test compounds to different GABA_A_-receptor subtypes. In recombinantly expressed receptors with a single α and a single β isoform, we expected to find receptor subtypes, which are sensitive to our test compound and 3H-muscimol binding can be completely displaced, and other subtypes, which are insensitive with no 3H-muscimol displacement. Surprisingly, this is not what we observed. Only our reference compound bicuculline seems to bind to all muscimol sites present in both brain derived and recombinantly expressed subunit combinations. Loxapine and clozapine, although showing more 3H-muscimol competition in α4β2-transfected HEK-293 cells, gave highly varying, incomplete displacement in all tested recombinant subunit combinations, indicating a complex mixed pharmacology even in our transfected cells. It is well known that in heterologous expression systems complex receptors formed from three or more subunits might assemble insufficiently and/or with multiple subunit arrangements, which complicates data interpretation (Ebert et al., 1996; Baumann et al., 2001). But even in cells, which are only transfected with one α- and one β-subtype, multiple receptor isoforms with different stoichiometries might be formed. Recombinantly expressed binary GABA receptors, composed of α and β subunits only, are traditionally assumed to mostly be composed of three β and two α subunits, here abbreviated as (β)_3_(α)_2_ (Tretter et al., 1997; Baumann et al., 2001), but other stoichiometric arrangements are possible. Since β subunits readily from homo-oligomers in heterologous expression systems (Slany et al., 1995; Miller and Aricescu, 2014; Hoerbelt et al., 2016), we assume that those receptors are also formed in our experiments, as well as receptors with four β and one α subunit. We therefore expect the following receptor subtypes (β2)_5_; (β2)_4_(α)_1_; (β2)_3_(α)_2_; and maybe even (β2)_2_(α)_3_ which might be formed in different ratios depending on yet unknown experimental conditions. Although independent from the stoichiometry, in cells that have been transfected with a single α- and a single β-isoform only one β+/α-interface can be formed, there could still be a heterogeneity of binding sites, since the individual properties of the orthosteric GABA_A_ binding site is also based on it’s second neighbor (Baumann et al., 2003). It therefore seems, that our three study compounds not only differentiate between different β2+/αx-interfaces, but also either between different β/α-stoichiometric arrangements, or between non-equivalent binding sites on a single pentamer.

We aimed to generate structural hypotheses for the differential ability of LOX, CLZ and CPZ to compete with muscimol and for the lower number of sites compared to bicuculline. A purely ligand based *in silico* analysis fails to provide a structural hypothesis for the differential displacements of 3H-muscimol by LOX and CLZ, but offers a plausible model for the low number of CPZ sensitive sites. Computational docking, while prone to give false positive solutions readily, provides some evidence for differential overlap of LOX and CLZ with bicuculline. Specifically, LOX can occupy the same volume in the 6HUK pocket as bicuculline, which places it at a distance to the variable segment (“loop”) F of the pocket and thus is consistent with the high overlap of LOX sensitive sites with the unselective BIC binding. CLZ in contrast is predicted to occupy a position in the pocket only partially overlapping with BIC, and closer to the variable segment F. These structural hypotheses provide the basis for further testing of other derivatives of LOX and CLZ as the base for the design of selective ligands for the 18 canonical orthosteric GABA/muscimol/bicuculline sites of the large family of GABA_A_ receptors. Future work can design slightly larger ligands based on our docking results, and use these alongside with point mutations to further refine bound state structural models, which is a commonly used approach to design selective ligands.

In total, this study provides evidence for heterogeneity of muscimol sites that can potentially be targeted with some selectivity, albeit low apparent affinities, by our tricyclic compounds. An obvious limitation beyond the high compound concentrations is the experimental variability that we observed, which appears to be of complex origin. In brain tissues, a certain degree of variability can be expected due to varying expression levels of the subunits, but the variability we observed in the recombinantly expressed subunit combinations points to additional factors as discussed for variable pools of receptors with different subunit stoichiometries and/or arrangements, and differential affinities for structurally or functionally non-equivalent β+/α-interfaces. Technical issues due to unfavourable kinetics and other factors also cannot be excluded. Future research thus should be aimed at further simplified and standardized receptor preparation to correlate the observed pools of loxapine, clozapine and chlorpromazine sensitive muscimol sites with specific β+/α-interfaces in defined pentameric assemblies, such as with the help of concatenated receptors (Baumann et al., 2001). In the long term, the development of genuinely selective orthosteric ligands based on loxapine, clozapine and suitable derivatives would be beneficial for many scientific and medical purposes.

## Data Availability

The raw data supporting the conclusion of this article will be made available by the authors, without undue reservation.
