# Supplementary material for: Probes for the heterogeneity of muscimol binding sites in rat brain
Source: Front Pharmacol. 2024 Mar 14;15:1368527. doi: 10.3389/fphar.2024.1368527 (PMC10973165; doi:10.3389/fphar.2024.1368527)
Supplement: Supplementary file 1 [file DataSheet1.docx]

Supplementary information

Probes for the Heterogeneity of Muscimol Binding Sites in Rat Brain

Veronika Müller^1†^, Margot Ernst^1†^, Aygul Baykuchkarova^1^, Filip Koniuszewski^1^, Konstantina Bampali^1^, Thomas Seidel^2^, Petra Scholze^1^*

†These authors contributed equally to this work and share first authorship

^1^Department of Pathobiology of the Nervous System, Center for Brain Research, Medical University of Vienna, Austria

^2^Department of Pharmaceutical Sciences, Division of Pharmaceutical Chemistry, University of Vienna, Vienna, Austria

*** Correspondence:**Petra Scholze
petra.scholze@meduniwien.ac.at

***SF1:*** ***Statistical analysis of the 3H-muscimol displacement at 1mM drug****.*

*Membranes were incubated with 10nM 3H-muscimol in the presence of 1 mM of drug. 100% is the amount of radioligand bound in the presence of 1% DMSO. Data shown are mean ± SEM of 3-9 independent experiments performed in triplicates each. One-way ANOVA followed by Tukey’s multiple comparisons test was performed to determine statistically significant differences between brain regions, where ns = p>0.05, *p<0.05, **p<0.01, ***p<0.001 and ****p<0.0001*

***SF2: Statistical analysis of the 3H-muscimol displacement data shown in Figure 2A.***

*Rat striatum membranes were incubated with 10nM 3H-muscimol in the presence of 1 mM of each drug. 100% is the amount of radioligand bound in the presence of 1% DMSO. Data shown are individual data points and mean values of 3-9 independent experiments performed in triplicates. One-way ANOVA followed by Tukey’s multiple comparisons test was performed to determine statistically significant differences between the different drug combinations, where ns = p>0.05, *p<0.05, **p<0.01, ***p<0.001 and ****p<0.0001*


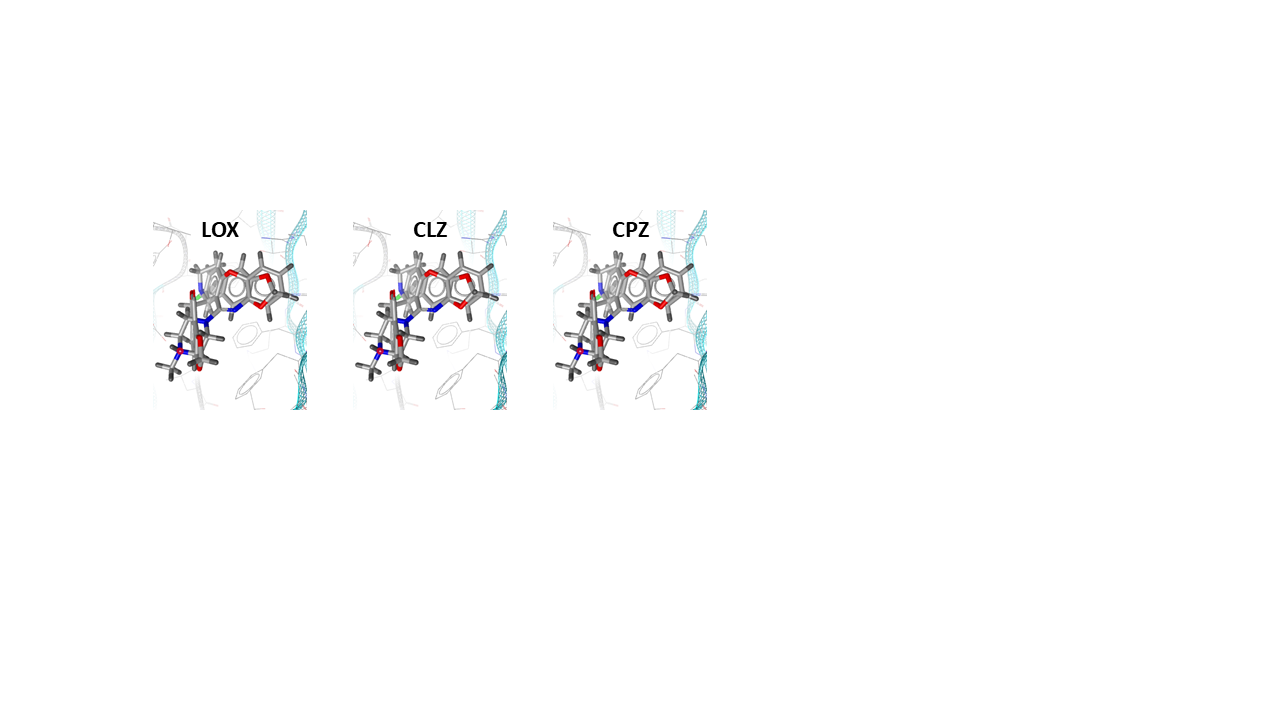


***SF 3: Shape alignment of BIC:*** *Bicuculline was aligned with LOX, CLZ and CPZ, respectively as described in the methods. All three alignments display large overlap and a surprising degree of shape similarity*


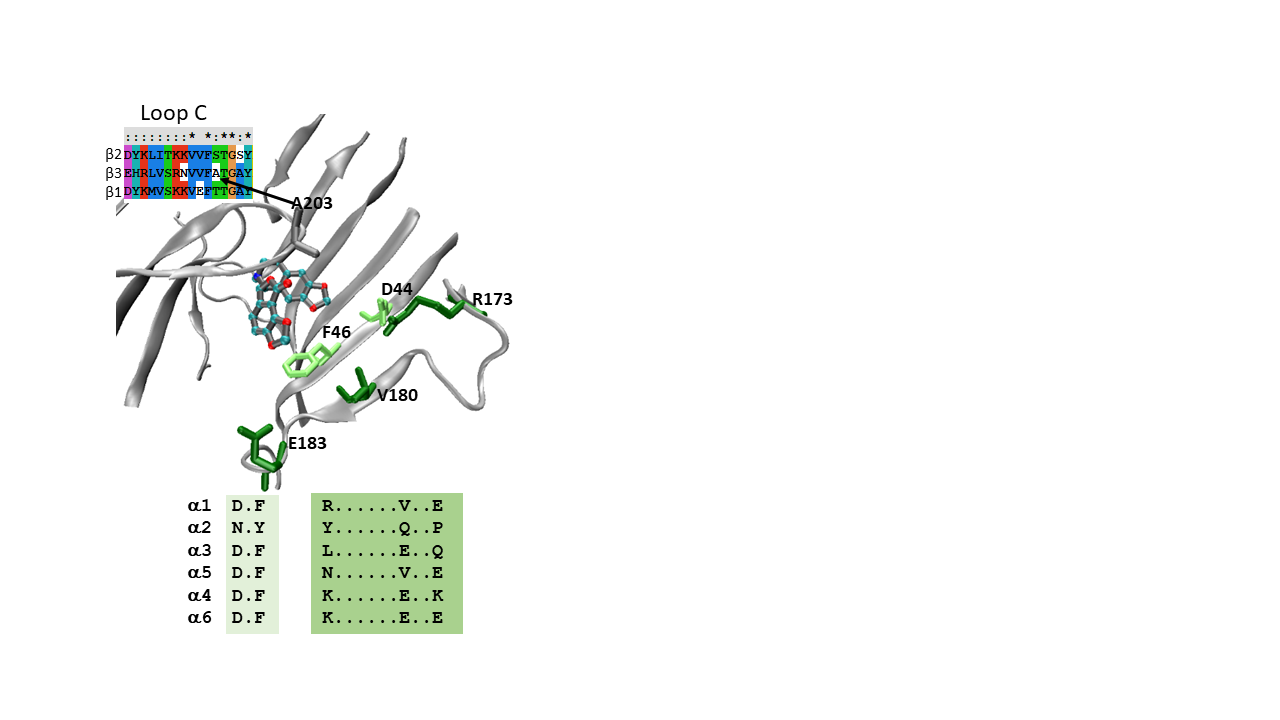


***SF 4: Variable pocket positions near the BIC pocket:*** *Rendering of 6HUK with variable amino acid positions highlighted. The upper insert displays the partial Clustal alignment of the three beta isoforms’ loop C region, the variable position which is A203 in 6HUK is marked with an arrow. The lower insert displays the variable positions of the six alpha isoforms on segments G (light green) and F (dark green) respectively. It can be seen that each alpha isoform has a unique constellation on segment F, which is not in direct contact with bicuculline but close enough to influence affinities of ligands that bind in a position closer to segment F, see Figure 4B.*
